# Development of Real-Time Cuffless Blood Pressure Measurement Systems with ECG Electrodes and a Microphone Using Pulse Transit Time (PTT)

**DOI:** 10.3390/s23031684

**Published:** 2023-02-03

**Authors:** Jingyu Choi, Younghwan Kang, Jaesoon Park, Yeunho Joung, Chiwan Koo

**Affiliations:** Department of Electronic Engineering, Hanbat National University, Daejeon 34158, Republic of Korea

**Keywords:** real-time, cuffless, blood pressure, pulse transit time, microphone

## Abstract

Research has shown that pulse transit time (PTT), which is the time delay between the electrocardiogram (ECG) signal and the signal from a photoplethysmogram (PPG) sensor, can be used to estimate systolic blood pressure (SBP) and diastolic blood pressure (DBP) without the need for a cuff. However, the LED of the PPG sensor requires the precise adjustment of both light intensity and light absorption rates according to the contact status of the light-receiving element. This results in the need for regular calibration. In this study, we propose a cuffless blood pressure monitor that measures real-time blood pressure using a microphone instead of a PPG sensor. The blood pulse wave is measured in the radial artery of the wrist using a microphone that can directly measure the sound generated by a body rather than sending energy inside the body and receiving a returning signal. Our blood pressure monitor uses the PTT between the R-peak of the ECG signal and two feature points of the blood pulse wave in the radial artery of the wrist. ECG electrodes and circuits were fabricated, and a commercial microelectromechanical system (MEMS) microphone was used as the microphone to measure blood pulses. The peak points of the blood pulse from the microphone were clear, so the estimated SBP and DBP could be obtained from each ECG pulse in real time, and the resulting estimations were similar to those made by a commercial cuff blood pressure monitor. Since neither the ECG electrodes nor the microphone requires calibration over time, the real-time cuffless blood pressure monitor does not require calibration. Using the developed device, blood pressure was measured three times daily for five days, and the mean absolute error (MAE) and standard deviation (SD) of the SBP and DBP were found to be 2.72 ± 3.42 mmHg and 2.29 ± 3.53 mmHg, respectively. As a preliminary study for proof-of-concept, these results were obtained from one subject. The next step will be a pilot study on a large number of subjects.

## 1. Introduction

Death due to cardiovascular disease can be prevented via disease management such as early diagnosis and appropriate monitoring. Monitoring certain physiological parameters such as heart rate, blood pressure, and pulse wave velocity helps prevent cardiovascular disease. Blood pressure is the pressure exerted by the blood flowing through the blood vessels on the walls of the blood vessels. High blood pressure typically causes the wall of one’s heart to thicken, and if it persists, it may lead to heart failure [1]. It also increases the risk of cerebral hemorrhage due to the dilation of blood vessels in the brain [2]. Since blood pressure can increase after a meal or change with a change in one’s posture, there is substantial demand for a healthcare product that can check for these changes in real time [3].

There are two types of methods used to measure blood pressure: invasive and non-invasive methods. The invasive method involves inserting a catheter directly into an artery and measuring blood pressure via a pressure transducer. This type of measurement is used when it is necessary to regularly monitor the blood pressure of an unconscious person. While the invasive method can accurately measure blood pressure, it is painful for the patient and risks wound infection. Meanwhile, non-invasive types, such as listening to Korotkoff sounds or sensing oscillometric pulses, measure blood flows through a person’s arteries. The Korotkoff sounds appear and disappear as blood flows or not when inflating or deflating a blood pressure cuff. By hearing it from the artery, medical persons can measure blood pressure from the patient. The oscillometric measurement uses an electronic pressure sensor to indirectly measure blood pressure, which can measure the oscillations of the artery by pressure variations. These non-invasive measurements have no risk of infection due to wounds, but the inconvenience involved in inflating the cuff represents a fundamental disadvantage, thereby limiting the continuous monitoring of blood pressure over a long-term period. A Holter monitor is a non-invasive blood pressure measurement device for continuously measuring blood pressure for a long period of time [4]. It allows patients to wear cuff blood pressure meters for 24 h and records changes in blood pressure while wearing them. However, since it still uses a cuff, patients may feel pain due to cuff expansion, and measurements can be inaccurate depending on the cuff’s size and location.

To solve the drawbacks of the cuff-based blood pressure measurement, many researchers published works using various approaches based on a pressure sensor [5,6], an electrocardiogram (ECG) [7,8,9], a pulse wave velocity (PWV) and a digital volume pulse (DVP) [10], and a pulse transit time (PTT) [11,12,13]. The blood pressure measurement using a pressure sensor proposed by Wang and Lin [14] requires the initial blood pressure, so it should be used along with a blood pressure monitor. The ECG confirmed that there is no strong correlation between the occurrence of hypertension and morphological changes in ECG [7]. The PWV uses the velocity at which blood pressure propagates through arteries, and the DVP uses the volume of the pulse wave. They mainly use photoplethysmogram (PPG) sensors, and specific coefficients are made using the systolic peak and diastolic peak of the signal. The blood pressure estimation method using PWV and DVP needs to detect four points using multiple sensors [10]. PTT is the time for blood to travel from the heart to other peripheral sites and is similar to PWV. Because the velocity is inversely related to time, PTT is inversely related to PWV. PTT uses fewer sensors than PWV and DVP, so it is simple to estimate blood pressure. Therefore, the PTT-based device is suitable for long-term continuous blood pressure monitoring, and it can be designed to be operated easily by the patient [15].

The existing PTT method uses an ECG sensor for the heart and a PPG sensor when measuring other peripheral parts. PPG can observe changes in blood flow by optically detecting light reflected or transmitted from tissues and blood. Based on the R-peak measured in the electrocardiogram, either the time difference between the start points of the pulse wave of the PPG signal or the time difference between the points when the PPG signal is used, and these have maximum values of PTTb and PTTt, respectively [16]. They are related to diastolic blood pressure (DBP) and systolic blood pressure (SBP), respectively. However, the PPG method requires the precise adjustment of the light intensity of the light-emitting device, and its light-receiving device must contact with the measurement site of the skin. Moreover, certain changes in blood flow and absorption of light occur due to the movement of the body, which limits the ability of the device to obtain accurate signals [17].

There are methods that can monitor body signals (abnormal symptoms) using sounds generated inside the body [18,19,20]. A representative example is using a stethoscope to analyze sounds generated from the heart to diagnose abnormalities in the heart. Among the acoustic methods, research measuring the sound generated in the body using a microphone is actively being conducted [21]. The microphones used in this study are mainly microelectromechanical system (MEMS) microphones, which are widely used in information technology (IT) fields such as in smartphones and wearable devices because they have a wide measurement frequency band, are inexpensive, and can be miniaturized. According to a study attempting to extract heart rates from the radial artery using a MEMS microphone, the PPG signal and the systolic and diastolic peaks of the microphone signal are correlated with each other [21]. The radial artery is relatively less affected by aging and blood pressure than other arteries, thus making it an ideal site for pulse rate evaluation [17]. Since the method using a MEMS microphone only measures signals generated inside the body rather than transmitting energy from outside and measuring signals reflected from the body, it is possible for this method to obtain more accurate measurements than the PPG method, which is an optical sensor method. However, a method for estimating blood pressure by combining a pulse wave measured by an acoustic method and an electrocardiogram has yet to be reported. Therefore, we propose a method for the real-time measurement of blood pressure using the PTT method with the electrocardiogram from the heart and the pulse wave generated from the radial artery of the wrist with a microphone. The developed ECG electrodes and microphone were used to read the time difference between the two signals. To evaluate our device, the measured blood pressures were compared to those obtained from a cuff-type blood pressure monitor certified as a medical device while changing the arm’s position, and repeated measurement experiments were conducted three times a day for five days to confirm reproducibility.

## 2. Materials and Methods

### 2.1. Pulse Transit Time (PTT)

**Figure 1:**
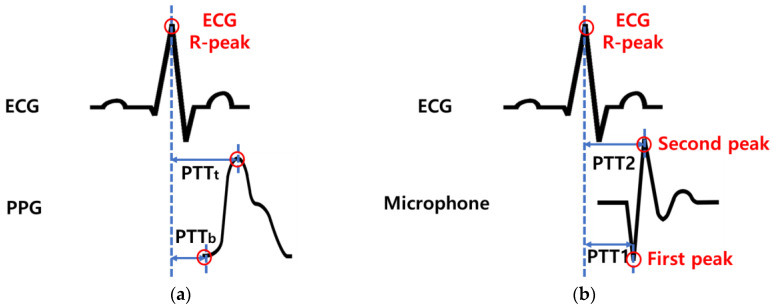
Principle of pulse transit time calculation. PTT_b_ and PPT1 are the time taken from the ECG R-peak to the first peak in the pulse wave. PTT_t_ and PTT2 are the time taken from the ECG R-peak to the second peak in the pulse wave. (**a**) When the PPG sensor is used and (**b**) when the microphone is used.

In the existing PTT method, the starting points of the pulse wave of the PPG signal measured in peripheral blood vessels such as in toes and fingers and the point in time having the maximum value are set as feature points. As shown in Figure 1a, the time difference between the time when the R-peak of the ECG occurs and the point at which the pulse wave of the PPG signal starts is PTTb, and the time difference between the time point when the pulse wave of the PPG signal has the maximum value is PTTt [16]. PTT has an inverse relationship with blood pressure, and PTTb and PTTt are related to diastolic blood pressure (DBP) and systolic blood pressure (SBP), respectively [16]. The waveform measured by the microphone sensor in the radial artery differs from the PPG waveform. The PPG sensor measures light that is reflected or transmitted from tissue and blood, while the microphone sensor measures changes in the membrane inside the sensor. The sound of blood flow generated in the radial artery makes the membrane vibrate and has a different form than the PPG pulse wave, as shown in Figure 1b [22].

The time points with the peak value in the microphone output waveform were set as the characteristic points. When the heart contracts and blood is pumped out at its maximum rate, the vibration of the membrane inside the microphone is maximized, so the first peak occurring in the negative direction is the maximum blood pressure point. The second peak occurring in the positive direction is the same as the peak point of PPG, so it is the diastolic blood pressure point [21]. Therefore, based on the R-peak of the ECG, the time differences when the first peak of the microphone signal is met and when the second peak of the microphone signal is met are PTT1 or PTT2, respectively.

Blood pressure is expressed in terms of kinetic energy and gravitational potential energy [23]. The distance, *d*, from the heart to the finger, the density of blood, *ρ*, the gravitational acceleration, *g*, and the height difference, *h*, between the two points to be measured can all be formulated mathematically as follows.

(1) $$\mathrm{BP}=\frac{\Delta \mathrm{BP}}{0.7}=\frac{1}{0.7}\left(\frac{1}{2}\rho \;\frac{d^{2}}{PTT^{2}}+\rho gh\right)=\frac{A}{PTT^{2}}+B$$

The average blood density *ρ* is 1035 kg/m^3^ [23]. In the formula, d may approximate the height of the subject. The distance between the two measured points is typically the distance from the heart to the finger, but in this paper, the same formula as Equation (2) was applied because it was instead measured from the heart to the wrist.

(2) $$A={\left(0.48\times height\;\right)}^{2}\times \frac{\rho }{1.4}\;$$

Equation (1) is corrected to reduce the uncertainty of the signal. In formula *A*, since there is no significant difference between subjects, the correction is only applied to *B* [23]. For the value for *B*, a fixed constant was used rather than writing the value accurately. In this experiment, about 40 mmHg is corrected.

### 2.2. Configuration of a Blood Pressure Measurement Device Using PTT

**Figure 2:**
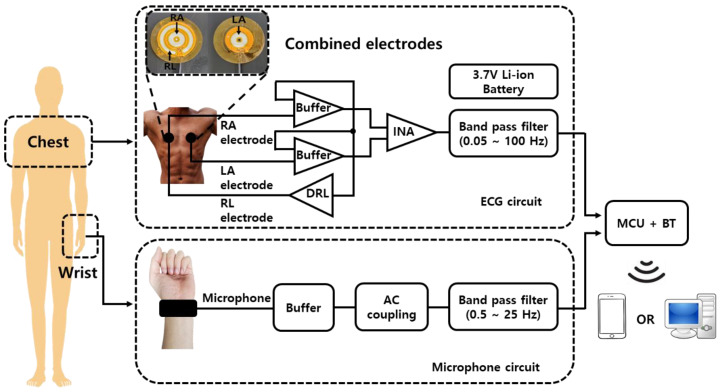
Block diagram of the developed real-time cuffless blood pressure measurement device using PTT.

The developed device measures ECG using electrodes attached to the chest, and it measures pulse waves using a microphone worn on the wrist. The electrodes and the microphone are connected to a separate wearable device, and the device consists of an ECG circuit for measuring the ECG and a microphone circuit for measuring the pulse wave (Figure 2). The ECG circuit consists of a 3.7 V Li-ion battery used as a power supply, electrodes to measure the electrocardiogram, an analog filter to remove noise, a microcontroller unit (MCU) to calculate blood pressure based on the measured signal, and a Bluetooth module to transmit data to an external device. The MCU performs 1 kHz sampling to obtain the ECG signal and the pulse wave signal, and then it calculates blood pressure.

#### 2.2.1. ECG Measurement Device

Existing ECG electrodes are wet electrodes that can obtain bio-signals without having a complex circuit design by using conductive ointments between the skin and electrode, but they lead to skin rash problems when used for a long time due to the denaturation of conductive ointment. To compensate for these problems, dry electrodes and capacitive electrodes that can measure bio-signals without conductive ointment have been developed. However, dry electrodes also have skin rash problems caused by metal. Capacitive electrodes form a dielectric layer by air with a high permittivity between the skin and the electrodes, and bio-signals are measured via capacitive coupling between the skin and electrodes. Capacitive electrodes have the advantages of being less affected by dynamic noise and involving less risk of skin rashes than dry electrodes [24]. We developed a capacitive electrode using copper film and silicon dioxide (SiO_2_) [25]. Copper, which is inexpensive and easy to process, was selected as the electrode material, and silicon dioxide, which has high dielectric strength, was used as a dielectric to increase the amount of charge accumulated in the electrode. When electrodes were attached to the chest and clothes are worn, triboelectricity is generated due to the movement between electrodes and clothes. To reduce this effect, polyimide—which has electrical insulation properties—was attached to the back of the electrode so that the polyimide was positioned between the electrode and the clothing. In addition, the right leg (RL) electrode that removes the common mode voltage can be combined with the right arm (RA) electrode. The size of the electrode used in the experiment is 10 mm, and the size of the electrode combined with RA and RL is 16 mm. Compared to other electrodes, the capacitive electrode requires a buffer for impedance matching, because the impedance between the skin and the electrode is high. Furthermore, the ECG signal generated by the heart has a frequency band ranging from 0.05 Hz to 100 Hz, and it requires a process of amplification after being measured as a small signal in mV units [26]. An instrumentation amplifier (INA) was used to amplify the difference in the measured bio-signals, and frequency bands other than those ranging from 0.05 Hz to 100 Hz, which is the ECG signal, were removed using a bandpass filter. Moreover, a driven right leg (DRL) circuit was constructed to minimize the effect on circuit noise.

#### 2.2.2. Pulse Wave Measurement Device

**Figure 3:**
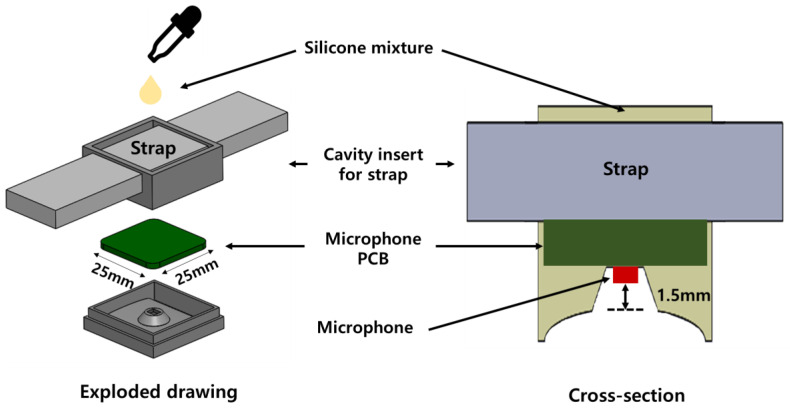
Schematic drawing of the wrist-type pulse wave device using a microphone (microphone device). The cavity insert for a strap is a space for a Velcro band. The distance between the microphone and the skin is 1.5 mm.

The selected microphone (SPW0442HR5H-1, KNOWLES, Itasca, IL, USA) is 3.1 mm × 2.5 mm × 1 mm in size, omnidirectional, and has a signal-to-noise ratio (SNR) of 59 dB(A) as well as a sensitivity of −42 dB(V) between 10 Hz and 10 kHz. The microphone type uses a capacitor, and the microphone converts the motion of the membrane into an electrical signal. The electrical signal is obtained by causing membrane movements inside the microphone using the sound of blood flow generated in the radial artery, which becomes a pulse wave. The signal waveform is important because it is necessary for distinguishing the characteristic points of the pulse wave generated from the radial artery. In this study, the amplification was set to 3 times. Since the noise is amplified as much as the signal is amplified, the main frequency component must be found using frequency analysis. As a result of the fast Fourier transform (FFT) analysis, the main frequency constituting the pulse wave generated in the radial artery was found to exist at 25 Hz, and a bandpass filter was used to remove components other than the main frequency [21]. The microphone circuit consists of AC coupling to remove DC, a bandpass filter to remove signals other than the main frequency, VCC, GND pads to receive power from the ECG circuit, and pads to send pulse wave signals to the ECG circuit. The microphone packaging was made in a wristwatch form that fixes the wrist and the structure. Its design was inspired by the shape of a bell, such as that used in a stethoscope to collect blood flow sounds generated from the radial artery (Figure 3). Silicone material was used so that the microphone’s housing could adhere well when in contact with the curved skin of the wrist. The part that contacts the skin is round in shape due to the shape of the wrist. Furthermore, a space for the watch strap to pass through is designed to fix the microphone packaging and the wrist. The polylactic acid (PLA) mold for manufacturing the silicone housing was made by using a 3D printer, placing a 25 mm × 25 mm × 1.6 mm microphone circuit board into the PLA mold, and pouring silicon. To remove noise from vibration from the skin, the distance between the microphone and the skin was set to 1.5 mm.

### 2.3. Performance Evaluation of Blood Pressure Measurement Device

**Figure 4:**
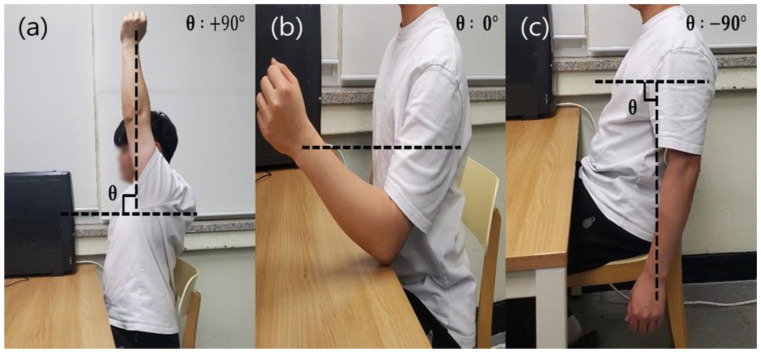
Three different arm positions for blood pressure measurement evaluation: (**a**) higher than the heart, (**b**) heart level, and (**c**) lower than the heart.

The ECG and the pulse wave signal measured by the manufactured device were analyzed. The microphone device was attached on the wrist of the left hand, and two ECG electrodes were attached on the chest. The MCU circuit for calculating blood pressure from the measured PTT was placed near the ECG. After the calculation, the results were compared with those obtained by a cuff blood pressure monitor that is certified as a medical device. This commercial monitor was placed on the right arm. All experiments were measured when the participants were sitting in a chair. Since this experiment aimed to confirm the possibility of measuring blood pressure by using ECG and pulse wave signals with two ECG electrodes and a microphone, it was conducted with one person. To evaluate the reproducibility of the measurement, it was repeated three times a day over five days. Before that, it was important to determine the best position of the microphone device on the wrist to obtain the most accurate blood pressure measurements possible. In the case of wrist cuff-type blood pressure monitors certified as medical devices, the standard practice is to use it at the same level as the heart. In the case of brachial cuff-type blood pressure monitors, the cuff’s position is at the same level as a heart. Since the developed device uses the microphone device on a wrist, it is necessary to check whether accurate blood pressure can be obtained at a specific arm height. The height of the arm was divided into three conditions: (a) higher than the heart, (b) at heart level, and (c) lower than the heart (Figure 4). Five measurements were taken at 1 min each (Figure 4). To compare the accuracy of the estimated blood pressure, a medical device-certified brachial cuff-type blood pressure monitor (HEM-7120, OMRON) and wrist cuff-type blood pressure monitor (HEM-6161, OMRON) were used.

Then, blood pressure was measured for five days to evaluate the measurement reproducibility of the developed device. The arm’s height was measured at the heart’s level, which is the standard practice for a cuff blood pressure monitor. Measurements were taken three times a day at 09:00, 15:00, and 21:00. A conventional brachial cuff-type blood pressure monitor was used every 2 min for 20 min, and 11 measurements were obtained. Then, our device was used for 20 min, and we calculated the mean absolute error (MAE) with the measurements.

## 3. Results and Discussions

### 3.1. Fabrication Results of Blood Pressure Measurement Device

**Figure 5:**
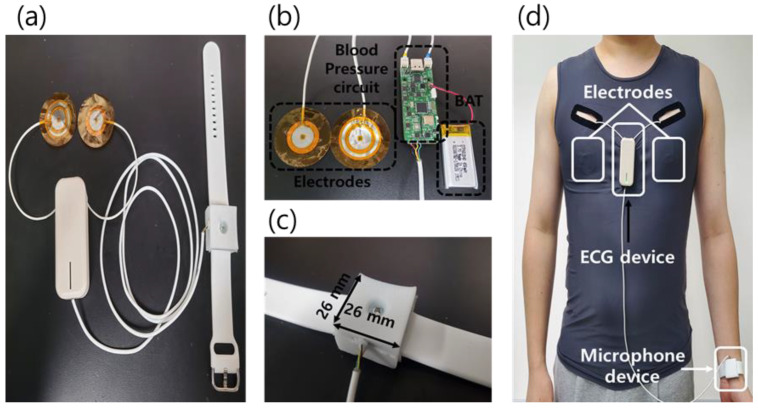
(**a**) The fabricated real-time cuffless blood pressure device. (**b**) The ECG device consists of two electrodes, an ECG circuit and an MCU, and a 3.7 V Li-ion battery. (**c**) The microphone device is connected to the ECG device with a thin electric wire to transmit the pulse wave signal to the MCU in the ECG device. (**d**) Picture of a participant wearing the blood pressure device.

The ECG device cover is designed so that it can make measurements by hanging it on one’s clothes, as the electrodes must be attached to the chest. Since the microphone device needs to receive power from the Li-ion battery of the ECG device without a separate power supply, the two circuits are connected with a line (Figure 5a,b). The microphone cover is round and contacts with the skin that is tailored to the shape of the wrist, and a watch strap is used to fix the microphone cover in place on the wrist. The size of the microphone cover is 26 × 26 × 15 mm^3^ (Figure 5c). The developed device was tested by attaching electrodes to the chest and a microphone device to the wrist (Figure 5d).

### 3.2. Performance Evaluation

**Figure 6:**
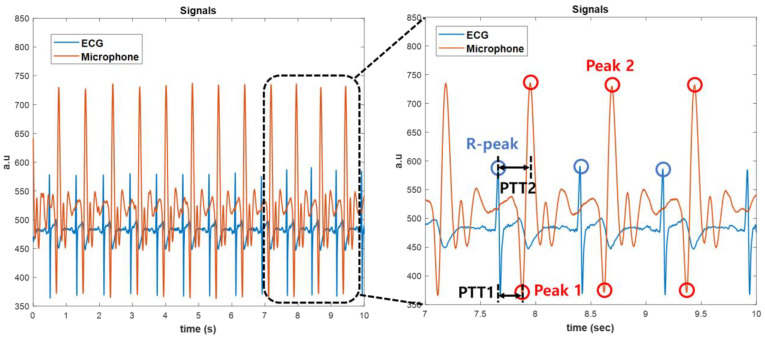
Simultaneous measurement of ECG and pulse wave. Distinction between an ECG R-peak and microphone peak points.

Based on a check with a PC using Bluetooth, the QRS wave of the ECG was measured with remarkable accuracy, and it was possible to achieve the R-peak detection of the ECG that was necessary for obtaining the PTT (Figure 6). The pulse wave with the microphone had an amplitude of about 1.1 V, and the first and second peaks were conspicuously confirmed in the noise-reduced signal. In the case of Figure 6, PTT1 and PTT2 were 222 msec and 292 msec, respectively. Furthermore, SBP and DBP were 113 mmHg and 83 mmHg, respectively, according to Equation (1). The results of the brachial cuff-type blood pressure monitor were 116 mmHg and 80 mmHg for SBP and DBP, respectively, and we were able to check the accuracy of results within about ± 4%.

**Figure 7:**
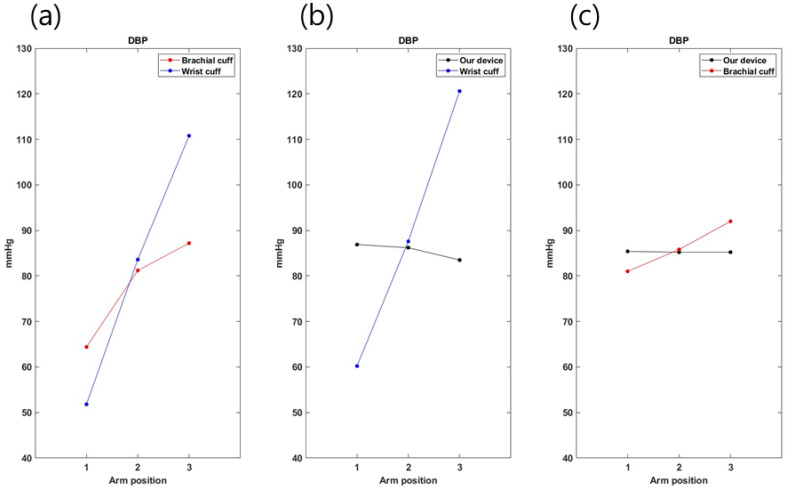
DBP measurement according to arm positions. (**a**) Comparison of two commercial cuff blood pressure monitors. (**b**) Comparison between our device and a wrist-type blood pressure monitor. (**c**) Comparison between our device and upper-arm-type cuff blood pressure monitor.

**Figure 8:**
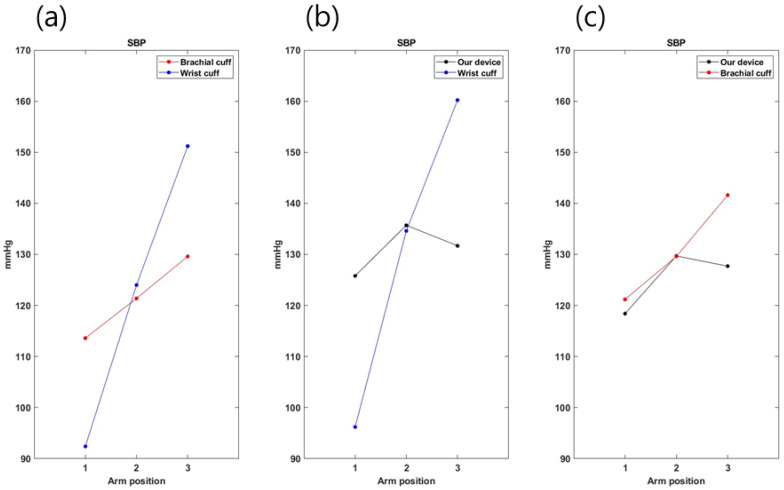
SBP measurement according to arm positions. (**a**) Comparison of two commercial cuff blood pressure monitors. (**b**) Comparison between our device and a wrist-type blood pressure monitor. (**c**) Comparison between our device and an upper-arm-type cuff blood pressure monitor.

To confirm the accuracy of the developed device by arm height, a comparative experiment was conducted for three heights. The experimental results were calculated as the average of five measurements (Figure 7 and Figure 8). First, in comparing the brachial cuff-type and the wrist cuff-type, both DBP and SBP were found to result in increased blood pressure with decreasing arm heights (1->2->3), and the results for height (2) were similar to each other. In the results of the developed device and the wrist cuff-type, both DBP and SBP showed similar results at arm height 2. Moreover, the developed device and upper arm cuff type results showed similar results at arm height 2 for both DBP and SBP. Therefore, the development device must be placed at the heart’s height to obtain similar results relative to the existing blood pressure monitor. Reflecting on the results from the previous experiment, the next experiment was measured at heart height.

**Figure 9:**
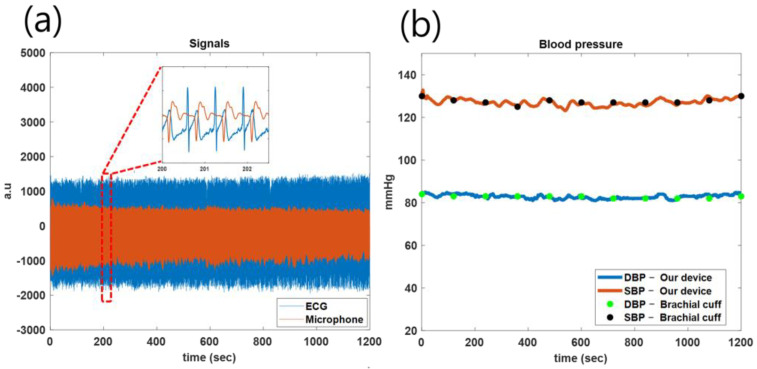
(**a**) ECG and microphone signals from a development device for 20 min measurement. (**b**) Reproducibility evaluation for the development device for 20 min measurement, compared to an arm upper cuff blood pressure monitor.

To evaluate the reproducibility of the developed device, blood pressure was measured three times a day at the same time for five days at heart height. Figure 9a shows a signal measured in real-time for 20 min. In Figure 9b, the results measured by the brachial cuff-type blood pressure monitor are displayed as dots, and the results measured by the developed device are displayed as lines. It can be observed that almost all line graphs pass through the points, and the results of the developed device are similar to those of the existing blood pressure monitor. The developed device can measure blood pressure in real-time at approximately once per second, based on a heart rate of 60. This is an improvement over existing blood pressure monitors, which measure blood pressure approximately once per minute, thus making real-time measurement impossible.

To evaluate the accuracy of the blood pressure measured by the device, the MAE and SD were obtained according to Equations (3) and (4), respectively:

(3) $$\mathrm{MAE}=\frac{\sum_{i=1}^{n}\left|y_{i}-x_{i}\right|}{n}$$

(4) $$\mathrm{SD}=\sqrt{\;\frac{\sum_{i=1}^{n}{\left(y_{i}-x_{i}-MAE\right)}^{2}}{n-1}}$$

where *x_i_* is the value measured by an automatic electronic blood pressure monitor (HEM-7120, OMRON), and *y_i_* is the blood pressure result obtained for n measurements on the device. As a result of the experiment, the average MAE ± SD of DBP and SBP were found to be 2.72 ± 3.42 mmHg and 2.29 ± 3.53 mmHg, respectively (Table 1). Considering the characteristics of blood pressure, which is sensitive to rapid changes in the surrounding environment or the body, it seems that the blood pressure results measured in real-time at a certain time every day using the developed device were similar to those obtained using cuff-type blood pressure monitors. In addition, the results shown in Table 1 were obtained without the need for additional daily calibration. According to the Association for the Advancement of Medical Instrumentation (AAMI), the accuracy of blood pressure is less than ±5 mmHg on average and the standard deviation of difference is less than 8 mmHg [27]; therefore, the measured results met the standard. However, since the results in Table 1 do not comprise data measured from multiple people, there is a limitation in that it cannot be confirmed whether blood pressure varies from person to person. In Equation (1), d is calculated by multiplying the height by a constant, but there will likely be variations among individuals, and the range of error values in blood pressure results caused by this deviation will be studied using clinical trials targeting a large number of subjects in the future. There is also a limitation in that comparing the result values of the cuff-type blood pressure monitor and the developed device, which cannot be measured in real-time, may not be an accurate comparison. These results should thus be compared with those obtained with the invasive method using a catheter, which is known to be able to measure blood pressure most accurately. After clinical trials on a large number of subjects, a comparative experiment with an invasive method will be conducted to confirm the accuracy of the developed device.

Lazazzera et al. presented a smartwatch made by CareUp^®^ (Farasha Labs, Paris, France) that calculates PTT using ECG and PPG and that estimates blood pressure using a linear model [28]. The experimental results obtained the SBP and DBP of 1.52 ± 9.45 mmHg and 0.39 ± 4.93 mmHg, respectively. Simjanoska et al. presented a machine learning model for estimating blood pressure using only the electrocardiogram, and SBP and DBP were obtained at 7.72 ± 10.22 mmHg and 9.45 ± 10.03 mmHg, respectively [7]. Wang et al. calculated the PTT using a strain-based pulse wave sensor and a PPG, and they established a blood pressure estimation model [29]. As a result, SBP and DBP were obtained at 3.71 ± 3.06 mmHg and 5.44 ± 5.10 mmHg, respectively. Compared to the studies presented in Table 2, we used a microphone sensor and ECG, which are inexpensive and can only obtain signals from the body. Long-term blood pressure estimation was possible in real-time without daily calculation, and the accuracy was obtained within ± 3 mmHg as MAE.

## 4. Conclusions

This study evaluated the possibility of measuring blood pressure after calculating PTT using an ECG from the heart and a pulse wave from the radial artery while utilizing two ECG electrodes and a microphone. It was possible to accurately measure blood pressure by distinguishing feature points of the pulse wave on the wrist with the microphone. The developed device does not require the initial blood pressure value of the blood pressure monitor for calibration, and it can continuously measure instantaneous blood pressure. The measured blood pressures were similar to the results obtained from commercial blood pressure cuffs that are certified as medical devices. To evaluate reproducibility, 15 measurements were taken over five days at the same time each day, and the MAE ± SD values of DBP and SBP were 2.72 ± 3.42 mmHg and 2.29 ± 3.53 mmHg, respectively. These results were obtained from one subject for a preliminary study, and the next step will be a pilot study involving a large number of subjects to demonstrate that this method can be a candidate for continuous long-term blood pressure measurements. 

## Figures and Tables

**Table 1:** Reproducibility evaluation for our device. Measurements taken three times a day for five days.

Day	Time	DBP Error (MAE ± SD mmHg)	SBP Error (MAE ± SD mmHg)
Day 1	09:00	2.23 ± 3.17	2.12 ± 2.42
	15:00	3.10 ± 0.71	2.55 ± 3.54
	21:00	2.95 ± 1.85	1.92 ± 5.90
Day 2	09:00	3.60 ± 0.084	1.67 ± 7.38
	15:00	2.17 ± 0.78	2.16 ± 2.93
	21:00	2.98 ± 2.35	1.75 ± 2.20
Day 3	09:00	2.89 ± 6.04	2.98 ± 2.25
	15:00	3.25 ± 6.23	3.18 ± 5.61
	21:00	2.65 ± 3.88	2.20 ± 5.32
Day 4	09:00	2.81 ± 5.65	3.05 ± 2.42
	15:00	2.16 ± 3.76	1.98 ± 3.40
	21:00	2.05 ± 4.95	2.57 ± 2.82
Day 5	09:00	2.87 ± 1.76	1.39 ± 1.72
	15:00	2.90 ± 3.80	2.17 ± 2.07
	21:00	2.22 ± 5.48	2.71 ± 3.04
Average		2.72 ± 3.42	2.29 ± 3.53

**Table 2:** Recent works for blood pressure measurement based on pulse transit time.

Authors	Technique	Statistical Method	SBP Error (mmHg)	DBP Error (mmHg)	Ref
Lazazzera et al.	PTT (ECG, PPG)	MAE ± SD	1.52 ± 9.45	0.39 ± 4.93	[28]
Simjanoska et al.	PTT (ECG)	MAE ± SD	7.72 ± 10.22	9.45 ± 10.03	[7]
Wang et al.	PTT (Strain-based pulse wave sensor, PPG)	MAE ± SD	3.71 ± 3.06	5.44 ± 5.10	[29]
Present Work	PTT (ECG, Microphone)	MAE ± SD	2.72 ± 3.42	2.29 ±3.53	This work

## Data Availability

Not applicable.
